# Notes for Contributors

**DOI:** 10.1155/S1463924679000705

**Published:** 1979


In summary, it does not make sense to train scientists in automation without also educating the non-scientists who will be the decision makers. Only such education, which is not now available, will speed the final transition of automation from a laboratory curiosity to a working laboratory tool.

*Clarence H. Annett*
*The University of Kansas*
*Lawrence, Kansas 66045*
*U.S.A.*

*Following is a reply to Annett from D.S. Young*

The clinical laboratory director abrogates one of his responsibilities if he allows a hospital administrator to dictate the choice of instrumentation regardless of whether it is automated or not. The administrator is untrained in any facet of laboratory work and should not have to be concerned with selection of equipment. The solution to the problem of proper choice of instrumentation raised by Annett is not education of administrators in analytical techniques and automation but education of clinical chemists and laboratory directors in business management practices. Whether one likes it or not clinical laboratories and hospitals have to be run as businesses with decisions made not only on the basis of quality of service but also on their cost-effectiveness. Thus, any new instrumentation should be evaluated in terms of the quality of results produced and the cost per test performed. In presenting a case for new equipment the laboratory director should demonstrate the improved cost-effectiveness of the new instrument. The hospital administrator is more concerned with this than other factors and his final endorsement of the purchase will, to a large extent, be dictated by the reduction in costs that will be achieved by the new equipment. While many clinical chemists and clinical pathologists have not had proper training in workload recording and cost-accounting, most training programs in clinical chemistry and many medical residency programs do include such training. There are also several books that deal with clinical laboratory management and include discussions of cost-accounting. Laboratory directors should expect to have to justify recommended purchases and the justifications should be in a form that is readily understood by the individual who has the final say.

If a technician is trained in the typical hospital laboratory he will almost certainly be exposed to some type of automation as few hospital laboratories associated with technician or technologist training do not have some automated equipment. There is of course a large variety of automated instruments and it is impossible even in the largest center for an individual to have experience with every type of automated instrument. Individuals entering the field of medical technology recognize the strong influence of instrumentation in the field. Some are deterred by it and tend to gravitate away from clinical chemistry towards microbiology, blood banking and hematology even though automation has made considerable strides in the area of hematology. Those who enjoy working with machines have found no problems in most instances in adapting themselves from a manual instrument to a mechanized way of doing the same thing. Obviously there is an onus on a laboratory director to discuss with his staff the impact of introducing automation in a previously non-automated laboratory and to ensure that the staff is properly trained, if they have not previously used the same equipment. He has the same responsibility even if the equipment is not automated.

In summary, I do not believe that the problems discussed by Annett are real if laboratory directors are properly trained. If there is a need for action, it is in better training of laboratory directors.

*D.S. Young*
*Mayo Clinic,*
*Rochester,*
*Minnesota,*
*U.S.A.*

### Notes for Contributors

### Presentation of manuscripts

Manuscripts should be typed (double-spaced) on one side of the paper only and with generous margins. The title should be brief and informative avoiding the word "new" and its synonyms. The full list of authors with their affiliations and full address(es) should appear on the title page. On a separate sheet an abstract of no more than 150 words is required. This should succinctly describe the scope of the contribution and highlight significant findings or innovations. It should be written in a style which can easily be translated into French and German.

*The Concise Oxford Dictionary* and Fowler's *Modern English Usage* (both published by Oxford University Press) should be used as the standard for spelling and grammar. Abbreviations should be limited to those generally recognised, or where a frequently occuring term is abbreviated it should, in the first instance, be explained thus "flow injection analysis (FIA) ..." and the abbreviation used thereafter. Abbreviations, for standard measures and units should follow SI recommendations. There are various publications giving guidance on the use of SI units.

References should be indicated in the text by numerals following the author's name, i.e. Skeggs [6]. On a separate sheet of paper, list all references in numerical order thus:

[6] Skeggs, L.T., *American Journal of Clinical Pathology,* 1959, **28**, 311.

Note that journal titles are given in full. Where there is more than one author, the form Foreman et al. should be used in the text but all authors should be named in the list of references. When reference is made to a chapter in a book the reference should take the following form:

[7] Malmstadt, H.V. in "Topics in Automatic Chemistry" Ed. Stockwell P.B. and Foreman J.K. 1978 Horwood, Chichester, pp. 68-70.

Only work which has been published or has been accepted for publication should be cited. Avoid the citation of documents which are subject to restricted circulation, patent literature, unpublished work and personal communications. The latter can be mentioned in the text in parenthesis.

To illustrate a paper line diagrams are preferred to photographs. Photographs should only be used when they significantly add to the discussion. Diagrams, charts and graphs should be carefully drawn in black ink on stout card or heavy quality tracing paper. Most illustrations are reduced for publication; to allow for this originals should be between 16 and 36 cm wide (the depth must not exceed 50 cm). The lettering of diagrams should be sufficiently clear to withstand reduction. Except in the case of proper names, all lettering should be in lower case print. If photographs are used they must be supplied in the form of clear, unmounted, glossy, black and white prints. "Instant" photographs are not normally acceptable. All illustrations must be identified on the reverse showing the figure number and the author's name.

Each illustration should have a fully explanitory caption. Captions should be typed together on a separate sheet of paper; they must *not* be an inseparable part of the illustration.

